# Comparisons of magnetic charge and axial charge meson cloud distributions in the PCQM

**DOI:** 10.1038/s41598-017-08648-w

**Published:** 2017-08-15

**Authors:** X. Y. Liu, Z. J. Liu, K. Khosonthongkee, A. Limphirat, Y. Yan

**Affiliations:** 1 School of Mathematics and Physics, Bohai University, Jinzhou, 121013 China; 2 School of Physics and Center of Excellence in High Energy Physics and Astrophysics, Suranaree University of Technology, Nakhon Ratchasima, 30000 Thailand; 3 School of Science, Shenyang University of Technology, Shenyang, 110870 China

## Abstract

The meson cloud distributions in r-space are extracted from the nucleon electromagnetic and axial form factors which are derived in the perturbative chiral quark model. The theoretical results indicate that the magnetic charge and axial charge distributions of the three-quark core have the similar distributions in r-space, the magnetic charge distributions of the meson cloud and three-quark core are more or less in the same region and peak at distances of around 0.4 fm, which is in good agreement with the finding of works in the framework of chiral perturbation theory, but the axial charge meson cloud distributes mainly inside the three-quark core.

## Introduction

The meson cloud of the nucleon, undoubtedly, plays a relevant role in the study of low energy electroweak properties of the nucleon. The meson cloud model, where the nucleon is considered as a system of three valence quarks surrounded by a meson cloud^1–12^, has recently been employed to study the generalized parton distribution^13, 14^, nucleon electroweak form factors^15–21^, nucleon strangeness^22–25^, etc. In refs 26–28, meson cloud contributions to the neutron charge form factor have been studied and discussed in the meson cloud model, while the effects of the meson cloud on electromagnetic transitions have been estimated in refs 29–31. In our previous works^32, 33^, the electromagnetic and axial form factors as well as electroweak properties of octet baryons have been studied in the perturbative chiral quark model (PCQM) in the low energy region ${Q}^{2}\le 1$ GeV^2^. The theoretical results in the PCQM with the predetermined quark wave functions are in good agreement with the experimental data and lattice QCD values. In addition, ref. 33 reveals that the meson cloud plays an important role in the axial charge of octet baryons, contributing 30–40% to the total values, and the similar effects have been also observed in other frameworks^34, 35^.

**Figure 1:**
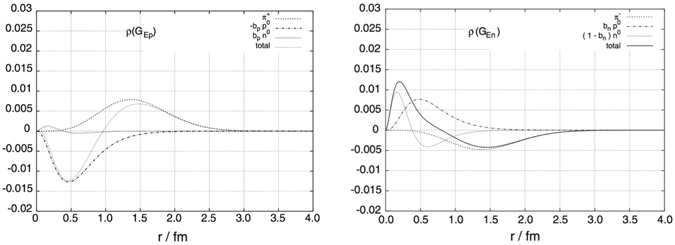
Shown as the dashed lines are the densities of nucleon charge contributed by the pion cloud, taken from ref. 36. Left panel: *r* ^2^ *ρ*(*r*) for the electric form factor of the proton. Right panel: *r* ^2^ *ρ*(*r*) for the electric form factor of the neutron.

**Figure 2:**
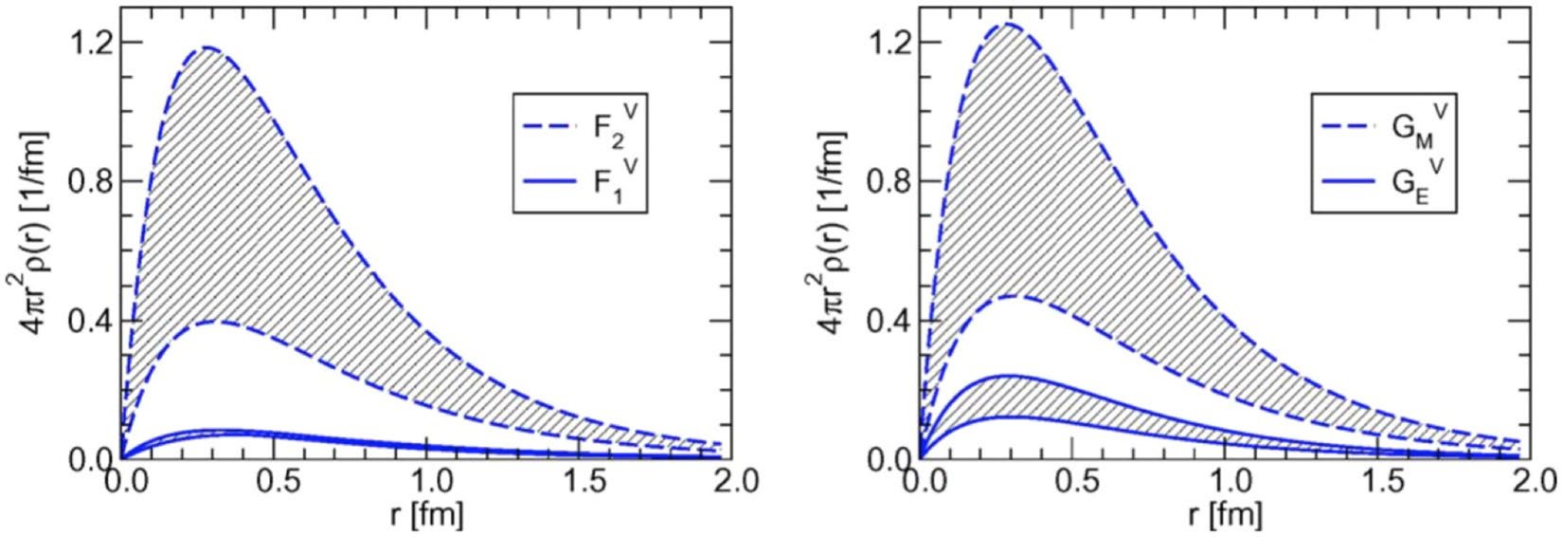
The densities of charge and magnetization from the pion cloud taken from ref. 37. Left panel: 4*πr* ^2^ *ρ*(*r*) for the isovector Pauli (upper band) and Dirac (lower band) form factors. Right panel: 4*πr* ^2^ *ρ*(*r*) for the isovector magnetic (upper band) and electric (lower band) Sachs form factors.

The investigation of the size or length scale of the meson cloud distribution inside the nucleon is interesting and important since it may help us to understand the in internal structure of nucleon intuitively. In ref. 36, the meson cloud distribution has been extracted from the nucleon EM form factors in the constituent quark model. The *π*-meson cloud distribution is found very long-ranged, ~2 fm (see Fig. 1 dashed curve), and is interpreted as the result of a pion cloud around the bare nucleon. Contrary to ref. 36, however, a much more confined *π*-meson cloud distribution of the nucleon EM form factors in *r*-space has been derived in the chiral perturbation theory (ChPT)^37, 38^. The results in refs 37 and 38 reveal that the *π*-meson cloud distributions peak around *r* = 0.3 fm and fall off smoothly with increasing the distance as shown in Fig. 2. Similar results have been also obtained in the chiral soliton model^39, 40^. The results in refs 37–40 may indicate that there is no structure at larger distances. In this work, we attempt to quantitatively study and define the *r*-space meson cloud distribution inside the nucleon in the framework of the PCQM.

## Perturbative chiral quark model

In the framework of the PCQM, baryons are considered as the bound states of three relativistic valence quarks moving in a central potential with *V*_eff_(*r*) = *S*(*r*) + *γ* ^0^ *V*(*r*), while a cloud of pseudoscalar mesons, as the sea-quark excitations, is introduced for chiral symmetry requirements, and the interactions between quarks and mesons are achieved by the nonlinear *σ* model in the PCQM. The Weinberg-type Lagrangian of the PCQM under an unitary chiral rotation^16, 17^ is derived as,

1 $${ {\mathcal L} }^{W}(x)={ {\mathcal L} }_{0}(x)+{ {\mathcal L} }_{I}^{W}(x)+o(\overrightarrow{\pi }),$$

2 $${{\mathcal{L}}}_{0}(x)=\bar{\psi }(x)[i{\rm{\partial }}/-{\gamma }^{0}V(r)-S(r)]\psi (x)-\frac{1}{2}{{\rm{\Phi }}}_{i}(x)(\square+{M}_{{\rm{\Phi }}}^{2}){{\rm{\Phi }}}^{i}(x),$$

3 $${ {\mathcal L} }_{I}^{W}(x)=\frac{1}{2F}{\partial }_{\mu }{{\rm{\Phi }}}_{i}(x)\bar{\psi }(x){\gamma }^{\mu }{\gamma }^{5}{\lambda }^{i}\psi (x)+\frac{{f}_{ijk}}{4{F}^{2}}{{\rm{\Phi }}}_{i}(x){\partial }_{\mu }{{\rm{\Phi }}}_{j}(x)\bar{\psi }(x){\gamma }^{\mu }{\lambda }_{k}\psi (x),$$

where *f*_*ijk*_ are the totally antisymmetric structure constant of *SU*(3), the pion decay constant *F* = 88 MeV in the chiral limit, Φ_*i*_ are the octet meson fields, and *ψ*(*x*) is the triplet of the *u*, *d*, and *s* quark fields taking the form

4 $$\psi (x)=(\begin{array}{c}u(x)\\ d(x)\\ s(x)\end{array}).$$

The quark field *ψ*(*x*) could be expanded in

5 $$\psi (x)=\sum _{\alpha }({b}_{\alpha }{u}_{\alpha }(\overrightarrow{x}){e}^{-i{ {\mathcal E} }_{\alpha }t}+{d}_{\alpha }^{\dagger }{\upsilon }_{\alpha }(\overrightarrow{x}){e}^{i{ {\mathcal E} }_{\alpha }t}),$$

where *b*_*α*_ and ${d}_{\alpha }^{\dagger }$ are the single quark annihilation and antiquark creation operators. The ground state quark wave function ${u}_{0}(\overrightarrow{x})$ may, in general, be expressed as

6 $${u}_{0}(\overrightarrow{x})=(\begin{array}{c}g(r)\\ i\overrightarrow{\sigma }\cdot \hat{x}f(r)\end{array}){\chi }_{s}{\chi }_{f}{\chi }_{c},$$

where *χ*_*s*_, *χ*_*f*_ and *χ*_*c*_ are the spin, flavor and color quark wave functions, respectively.

**Figure 3:**
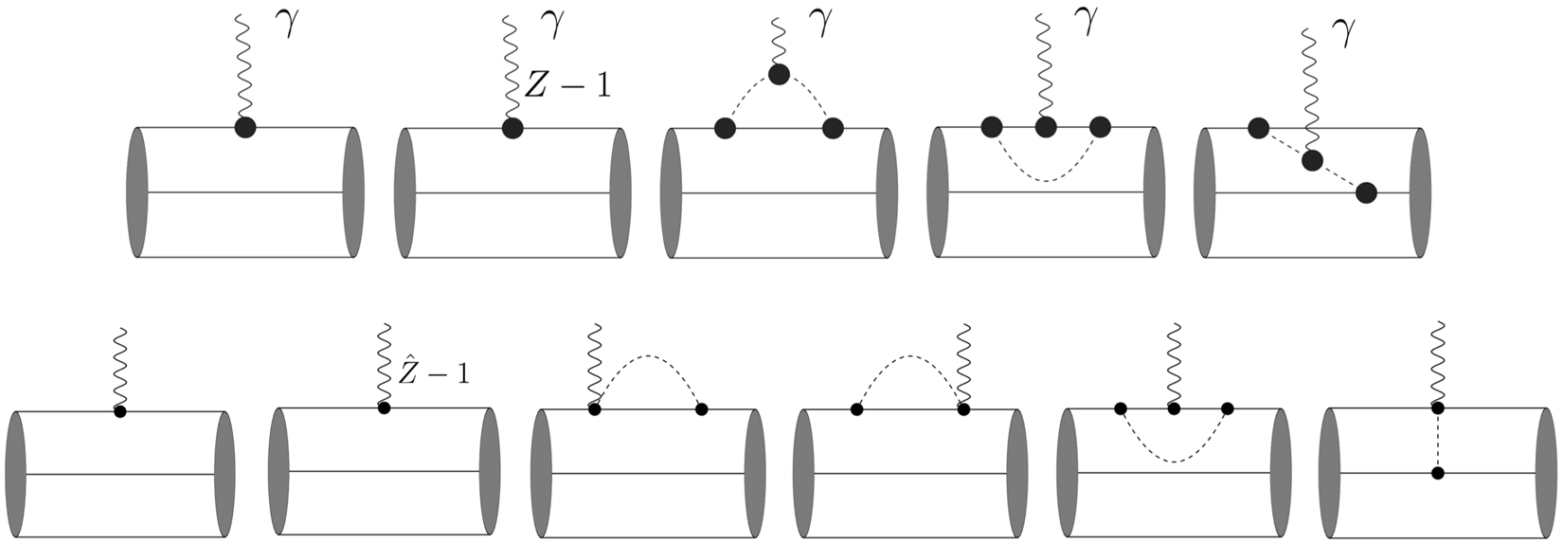
Feynman diagrams contributing to the octet baryons electromagnetic form factors (upper panel) and axial form factors (lower panel) up to one-loop order.

The calculation technique in the PCQM is based on the Gell-Mann and Low theorem^41^, in which the expectation value of an operator $\hat{O}$ can be calculated from

7 $$\langle \hat{O}\rangle ={}^{B}\langle {\varphi }_{0}|\sum _{n=0}^{\infty }\frac{{i}^{n}}{n!}\int {d}^{4}{x}_{1}\cdots \int {d}^{4}{x}_{n}T[{ {\mathcal L} }_{I}^{W}({x}_{1})\cdots { {\mathcal L} }_{I}^{W}({x}_{n})\hat{O}]{|{\varphi }_{0}\rangle }_{c}^{B},$$

where the state vector |*ϕ*_0_〉^*B*^ corresponds to the unperturbed three-quark states projected onto the respective baryon states, which are constructed in the framework of the *SU*(6) spin-flavor and *SU*(3) color symmetry. The subscript *c* in Eq. (7) refers to contributions from connected graphs only. ${ {\mathcal L} }_{I}^{W}(x)$ is the quark-meson interaction Lagrangian as given in Eq. (3). The Feynman diagrams contributing to the electromagnetic and axial form factor of octet baryons up to the one-loop order are shown in upper and lower panel of Fig. 3, respectively.

**Figure 4:**
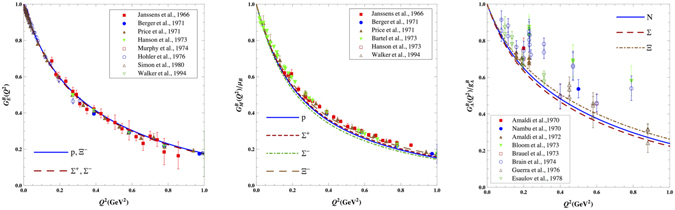
The results of EM and axial form factors in the PCQM taken from refs 32, 33.

In our previous works^32, 33^, the ground state quark wave functions have been determined by fitting the PCQM theoretical result of the proton charge form factor ${G}_{E}^{p}({Q}^{2})$ to the experimental data^32^, and the electromagnetic and axial form factors as well as electroweak properties of octet baryons in low energy region (${Q}^{2}\le 1$ GeV^2^) have been studied in the PCQM based on the predetermined quark wave functions. As the results shown in Fig. 4, the EM and axial form factors are in good agreement with the experimental data up to *Q* ^2^ = 1 GeV^2^ based on the predetermined quark wave functions. Meanwhile, the nucleon magnetic moment *μ*_*p*_ = 2.735 ± 0.121 and axial charge ${g}_{A}^{N}=1.301\pm 0.230$, which are the magnetic and axial form factors in zero-recoil, differ from the experimental data by only 2%, and are also consistent with the lattice QCD values. It is noted that there is no any free parameter in the numerical calculations. Thus one may indicate that the PCQM is credible for the low energy region ${Q}^{2}\le 1$ GeV^2^, and able to quantitatively study and evaluate the *r*-space meson cloud distribution in the frame work of the PCQM. More details could be found in refs 32 and 33.

### Magnetic and axial charge distributions of meson cloud

**Figure 5:**
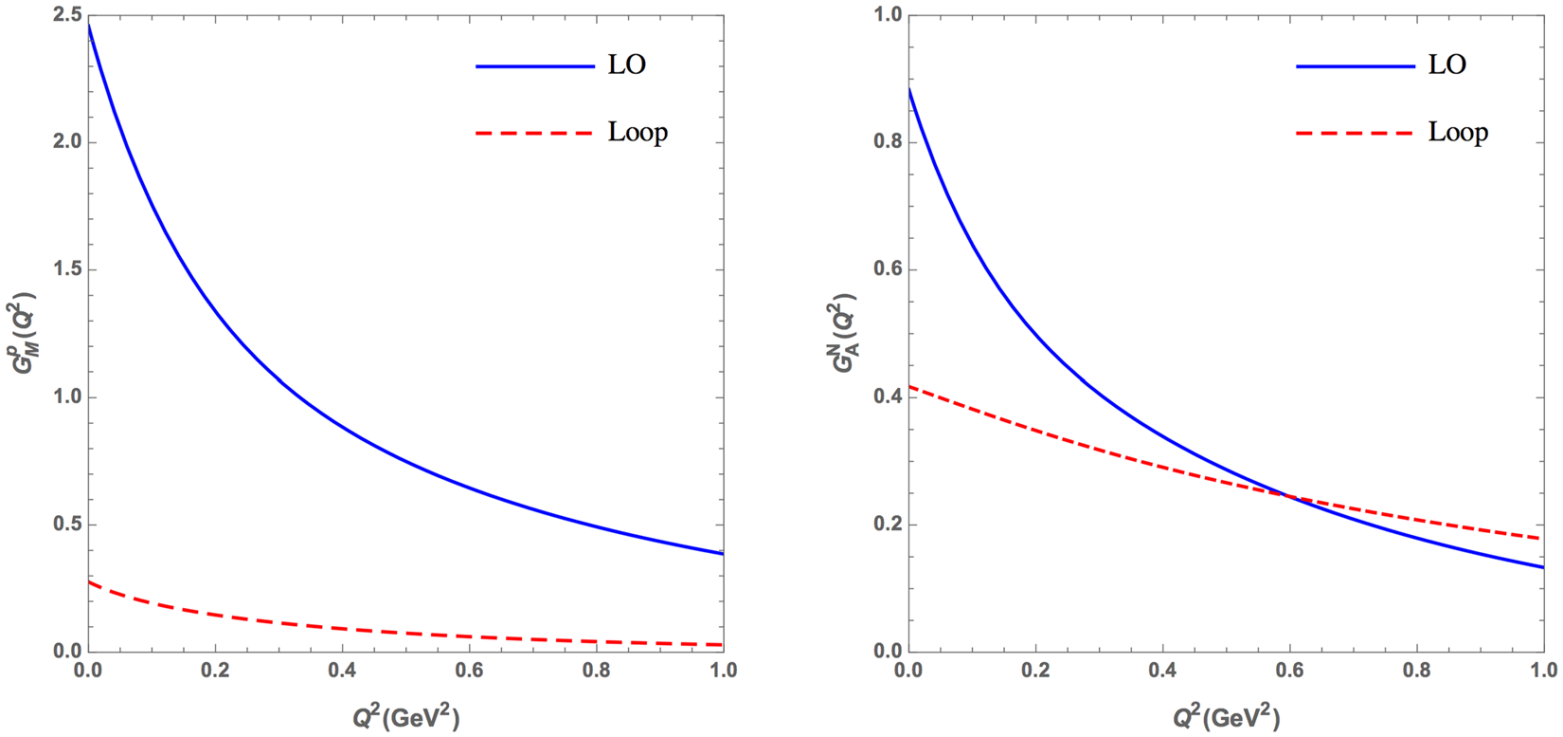
Leading order (solid) and loop (dashed) contributions to the proton magnetic (left panel) form factor and neutron axial (right panel) form factor.

Following our previous works^32, 33^, we present in Fig. 5 the *Q* ^2^-dependence of proton magnetic and nucleon axial form factors separately in leading order (LO) diagram and loop Feymann diagrams. This division is the PCQM dependent, and the LO diagram is attributed to 3q-core and the loop Feymann diagrams could be interpreted as the effects of the pion cloud. The PCQM results shown in Fig. 5 clearly reveal that the LO diagram results in a dipole-like form factor and dominates the form factor, while the meson cloud leads to a flat contribution to the magnetic and axial form factors. The flat contribution may indicate that the meson cloud of the nucleon may distribute mainly in a very small region.

In general, the form factor *F*(*q* ^2^) is the Fourier transformation of charge distribution in *r*-space and takes the form,

8 $$F({q}^{2})=\int \rho (\overrightarrow{r}){e}^{-i\overrightarrow{q}\cdot \overrightarrow{r}}{d}^{3}\overrightarrow{r},$$

where $\rho (\overrightarrow{r})$ is the charge density, and $\overrightarrow{q}$ is the three-momentum transfer. If *F*(*q* ^2^) has been determined, in principle, the charge distribution *ρ*(*r*) could be derived by the inverse Fourier transformation,

9 $$\rho (\overrightarrow{r})=\frac{1}{{\mathrm{(2}\pi )}^{3}}\int F({q}^{2}){e}^{i\overrightarrow{q}\cdot \overrightarrow{r}}{d}^{3}\overrightarrow{q}.$$

In this work, we extract, based on the inverse Fourier transformation Eq. (9), the magnetic charge and axial charge meson cloud distributions of the nucleon from the EM and axial form factors as shown in Fig. 5.

**Figure 6:**
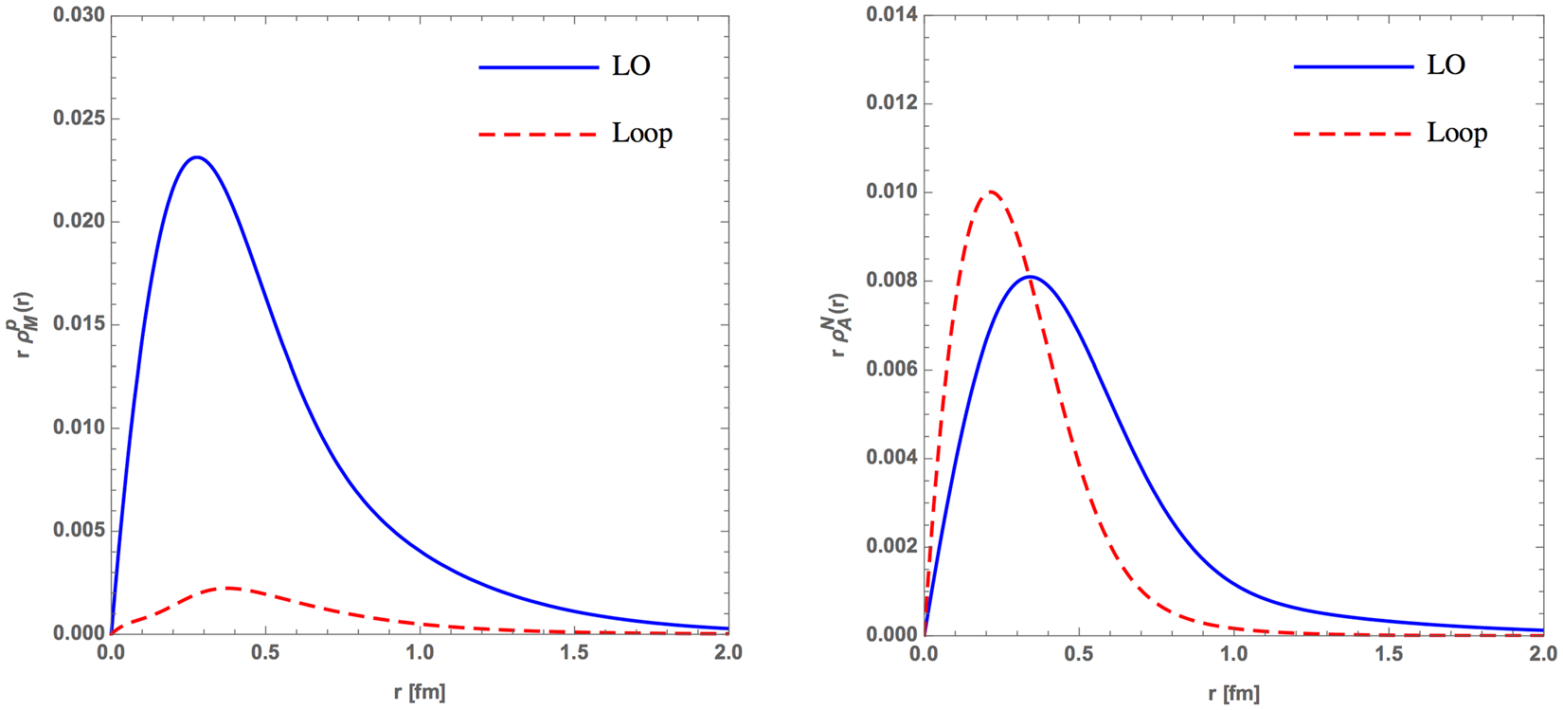
Comparisons between the LO and meson cloud distributions for proton magnetic (left panel) and axial (right panel) form factors in *r*-space.

The results shown in Fig. 6 are the LO and meson cloud contributions to the proton magnetic form factor ${\rho }_{M}^{p}(r)$ (left panel) and the nucleon axial form factor ${\rho }_{A}^{N}(r)$ (right panel) in *r*-space derived by Eq. (9). It is clear that the magnetic and axial charge attributed to 3q-core (LO) show a distribution ranging 2 fm, while the meson cloud effects (Loop) are much smaller at distances beyond 1 fm which is in accordance with the finding of ref. 37 as shown in Fig. 2. But it is much smaller than the result of ref. 36 about 2.5 fm (see Fig. 1), in which the proton is throught of as virtual neutron-positively charged pion pair. As results shown in the left panel of Fig. 6, the peaks of the ${\rho }_{M}^{p}(r)$ of the 3q-core and the meson cloud are almost in the same region, but the peak of the loop diagrams contributions to the ${\rho }_{A}^{N}(r)$ as presented in the right panel of Fig. 6 are in a clearly smaller region than the one of 3q-core, which may indicate that the axial charge meson cloud distributes mainly inside the three-quark core.

**Figure 7:**
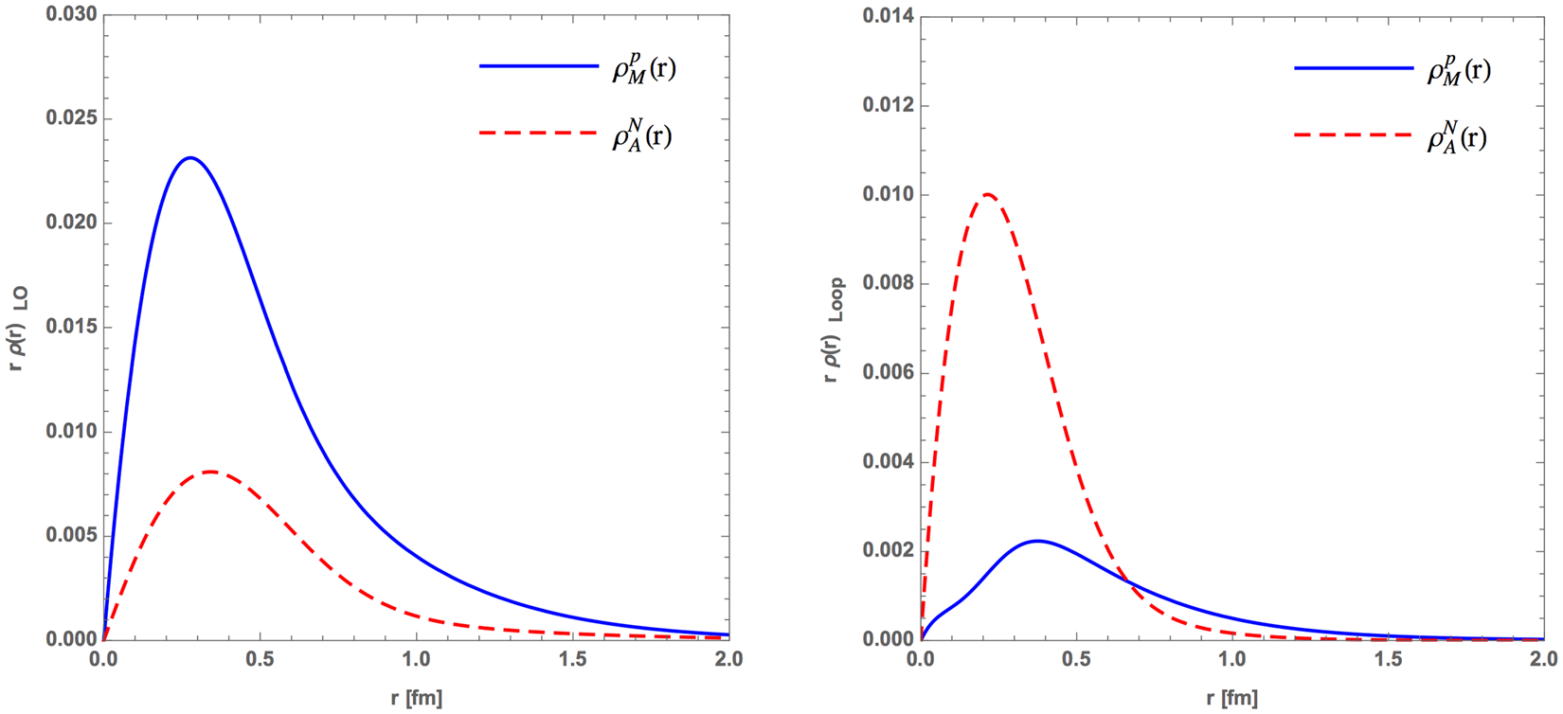
Comparisons between the magnetic and axial distributions in *r*-space for the LO (left panel) and loop (right panel) diagrams.

Furthermore, we compare the 3q-core contributions to the ${\rho }_{M}^{p}(r)$ and ${\rho }_{A}^{N}(r)$ in *r*-space as presented in the left panel of Fig. 7. It is found that the ${\rho }_{M}^{p}(r{)|}_{LO}$ and ${\rho }_{A}^{N}(r{)|}_{LO}$ show a similar *r*-dependence, which may reveal that the magnetic charge and axial charge distributions of the constituent quarks are the same. The meson cloud contributing to ${\rho }_{M}^{p}(r)$ and ${\rho }_{A}^{N}(r)$ in the right panel of Fig. 7 show that the axial charge distribution of the meson cloud ${\rho }_{A}^{N}(r{)|}_{Loop}$ is narrower and the peak is closer to the origin. We also found that the magnetic charge distribution ${\rho }_{M}^{p}(r{)|}_{Loop}$ in the right panel of Fig. 7 present a significant peak around $r\simeq 0.4$ fm and fall off smoothly when the distance increases. To compare with the right panel of Fig. 2, the ${\rho }_{M}^{p}(r){|}_{Loop}$ distribution in the PCQM turns out to be similar to the ChTP finding of refs 37, 38.

## Summary

In this work, we quantitatively study and evaluate the electromagnetic and axial form factors of nucleon distribution in *r*-space in the framework of the PCQM. The PCQM-dependent results are separated into the 3q-core contributions and the meson cloud effects, and the results are in good agreement with the ChPT results of refs 37, 38. In summary, one may conclude that the similar r-dependence of the magnetic and axial form factors resulted from the LO diagrams show a distribution ranging 2 fm and may reveal that the magnetic charge and axial charge distributions of the constituent quarks are the same. The meson cloud effects to the magnetic and axial charge distributions are much smaller at distances beyond 1 fm which is in accordance with the finding of refs 37, 38. Meanwhile, the magnetic charge distributions of the 3q-core and the meson cloud are more or less in the same region and peak at distances of around 0.4 fm, quite consistent with the earlier determinations of ChPT in refs 37 and 38, while the peak of the loop diagrams contributions to the ${\rho }_{A}^{N}(r)$ are in a clearly smaller region than the one of 3q-core, which may indicate that the axial charge meson cloud distributes mainly inside the 3q-core.

## References

[1] Théberge S, Thomas AW, Miller GA (1980). Pionic corrections to the mit bag model: The (3,3) resonance. Phys. Rev. D.

[2] Thomas AW, Théberge S, Miller GA (1981). Cloudy bag model of the nucleon. Phys. Rev. D.

[3] Chin S (1982). Many-body theory of confined quarks and excluded pions: A perturbative study of the chiral bag. Nucl. Phys. A.

[4] Oset E, Tegen R, Weise W (1984). Nucleon charge form factors and chiral quark models. Nucl. Phys. A.

[5] Gutsche T, Robson D (1989). Positive and convergent self-energy in a chiral potential model. Phys. Lett. B.

[6] Dziembowski Z, Holtmann H, Szczurek A, Speth J (1997). Pionic corrections to nucleon electromagnetic properties in a light-cone framework. Ann. Phys..

[7] Speth J, Thomas A (2002). Mesonic contributions to the spin and flavor structure of the nucleon. Adv. Nucl. Phys..

[8] Tiator L (2004). Electroproduction of nucleon resonances. Eur. Phys. J. A.

[9] Faessler A, Gutsche T, Lyubovitskij VE, Pumsa-ard K (2006). Chiral dynamics of baryons in a lorentz covariant quark model. Phys. Rev. D.

[10] Juliá-Díaz B, Riska D (2006). The role of components in the nucleon and the resonance. Nucl. Phys. A.

[11] Chen D, Dong Y, Giannini M, Santopinto E (2007). Hypercentral constituent quark model with a meson cloud. Nucl. Phys. A.

[12] Aznauryan IG (2013). Studies of nucleon resonance structure in exclusive meson electroproduction. Int. J. Mod. Phys. E.

[13] Pasquini B, Boffi S (2006). Virtual meson cloud of the nucleon and generalized parton distributions. Phys. Rev. D.

[14] Pasquini B, Boffi S (2007). Generalized parton distributions in a meson cloud model. Nucl. Phys. A.

[15] Lyubovitskij VE, Gutsche T, Faessler A (2001). Electromagnetic structure of the nucleon in the perturbative chiral quark model. Phys. Rev. C.

[16] Lyubovitskij V, Gutsche T, Faessler A, Mau RV (2001). *π* n scattering and electromagnetic corrections in the perturbative chiral quark model. Phys. Lett. B.

[17] Khosonthongkee K (2004). Axial form factor of the nucleon in the perturbative chiral quark model. J. Phys. G: Nucl. Part. Phys..

[18] Pasquini B, Boffi S (2007). Electroweak structure of the nucleon, meson cloud, and light-cone wave functions. Phys. Rev. D.

[19] Adamuščn C, Tomasi-Gustafsson E, Santopinto E, Bijker R (2008). Two-component model for the axial form factor of the nucleon. Phys. Rev. C.

[20] Ramalho G, Tsushima K (2011). Octet baryon electromagnetic form factors in a relativistic quark model. Phys. Rev. D.

[21] Ramalho G, Tsushima K, Thomas AW (2013). Octet baryon electromagnetic form factors in nuclear medium. J. Phys. G: Nucl. Part. Phys..

[22] Carvalho F, Navarra FS, Nielsen M (2005). Can the meson cloud explain the nucleon strangeness?. Phys. Rev. C.

[23] Bijker R, Santopinto E (2009). Unquenched quark model for baryons: Magnetic moments, spins, and orbital angular momenta. Phys. Rev. C.

[24] Chen H, Cao FG, Signal AI (2010). Strange sea distributions of the nucleon. J. Phys. G: Nucl. Part. Phys..

[25] Bijker R, Ferretti J, Santopinto E (2012). *s**s* sea pair contribution to electromagnetic observables of the proton in the unquenched quark model. Phys. Rev. C.

[26] Lu D, Tsushima K, Thomas A, Williams A, Saito K (1998). The neutron charge form factor in helium-3. Phys. Lett. B.

[27] Glozman L, Riska D (1999). Pionic fluctuations of constituent quarks and the neutron charge radius. Phys. Lett. B.

[28] Rinehimer JA, Miller GA (2009). Neutron charge density from simple pion cloud models. Phys. Rev. C.

[29] Ramalho G, Jido D, Tsushima K (2012). Valence quark and meson cloud contributions for the *γ**Λ → Λ* and *γ**∑^0^ → Λ* reactions. Phys. Rev. D.

[30] Ramalho G, Tsushima K (2013). What is the role of the meson cloud in the ∑^*0^ → *γ*Λ and ∑^*^ → *γ*∑ decays?. Phys. Rev. D.

[31] Ramalho G, Peña MT (2014). *γ***n* → *N** (1520) form factors in the spacelike region. Phys. Rev. D.

[32] Liu XY, Khosonthongkee K, Limphirat A, Yan Y (2014). Study of baryon octet electromagnetic form factors in perturbative chiral quark model. J. Phys. G: Nucl. Part. Phys..

[33] Liu XY, Khosonthongkee K, Limphirat A, Suebka P, Yan Y (2015). Meson cloud contributions to baryon axial form factors. Phys. Rev. D.

[34] Franklin J (2002). Phenomenological quark model for baryon magnetic moments and beta decay ratios (*G*_*A*_/*G*_*V*_). Phys. Rev. D.

[35] Ramalho, G. & Tsushima, K. Axial form factors of the octet baryons in a covariant quark model. *arXiv:1512.01167 [hep-ph]* (2016).

[36] Friedrich J, Walcher T (2003). A coherent interpretation of the form factors of the nucleon in terms of a pion cloud and constituent quarks. Eur. Phys. J. A.

[37] Hammer HW, Drechsel D, Meißner UG (2004). On the pion cloud of the nucleon. Phys. Lett. B.

[38] Meißner UG (2007). The pion cloud of the nucleon: Facts and popular fantasies. AIP Conf. Proc..

[39] Meißner UG (1988). Low-energy hadron physics from effective chiral lagrangians with vector mesons. Phys. Rep..

[40] Holzwarth G (1996). Electro-magnetic nucleon form factors and their spectral functions in soliton models. Z. Phys. A.

[41] Murray G, Francis L (1951). Bound states in quantum field theory. Phys. Rev..

